# Schwann Cell Role in Selectivity of Nerve Regeneration

**DOI:** 10.3390/cells9092131

**Published:** 2020-09-20

**Authors:** Sara Bolívar, Xavier Navarro, Esther Udina

**Affiliations:** Institute of Neurosciences, Department Cell Biology, Physiology and Immunology, Universitat Autònoma de Barcelona, and Centro de Investigación Biomédica en Red sobre Enfermedades Neurodegenerativas (CIBERNED), 08193 Bellaterra, Spain; sara.bolivar@uab.cat (S.B.); Xavier.Navarro@uab.cat (X.N.)

**Keywords:** axon, Schwann cell, regeneration, axon-glia interactions, peripheral nerve injury, reinnervation accuracy, preferential motor reinnervation, motor, sensory

## Abstract

Peripheral nerve injuries result in the loss of the motor, sensory and autonomic functions of the denervated segments of the body. Neurons can regenerate after peripheral axotomy, but inaccuracy in reinnervation causes a permanent loss of function that impairs complete recovery. Thus, understanding how regenerating axons respond to their environment and direct their growth is essential to improve the functional outcome of patients with nerve lesions. Schwann cells (SCs) play a crucial role in the regeneration process, but little is known about their contribution to specific reinnervation. Here, we review the mechanisms by which SCs can differentially influence the regeneration of motor and sensory axons. Mature SCs express modality-specific phenotypes that have been associated with the promotion of selective regeneration. These include molecular markers, such as L2/HNK-1 carbohydrate, which is differentially expressed in motor and sensory SCs, or the neurotrophic profile after denervation, which differs remarkably between SC modalities. Other important factors include several molecules implicated in axon-SC interaction. This cell–cell communication through adhesion (e.g., polysialic acid) and inhibitory molecules (e.g., MAG) contributes to guiding growing axons to their targets. As many of these factors can be modulated, further research will allow the design of new strategies to improve functional recovery after peripheral nerve injuries.

## 1. Introduction

**Figure 1:**
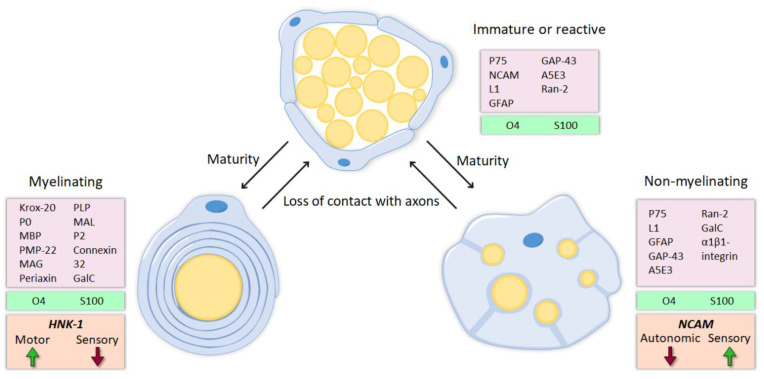
During development, immature SCs differentiate into myelinating or nonmyelinating, depending on the type of axon they ensheath. These two mature phenotypes are characterized by the expression of different markers, some of which are preferentially expressed on a modality-specific manner. Myelinating SCs that wrap motor axons express the carbohydrate HNK-1, whereas sensory SCs have low levels. NCAM is mostly expressed among nonmyelinating SCs, especially in those that ensheath sensory unmyelinated fibers. After nerve injury, due to the loss of contact with the axons, SCs downregulate molecules related to myelin and return to a regenerating phenotype which is very similar to the immature state.

Peripheral nerve injuries may have devastating consequences, as they result in the loss of the motor, sensory and autonomic functions of the part of the body innervated by the injured nerve or trunk. Following peripheral nerve injury, neurons are disconnected from their target organs and initiate a regenerative response. Axotomy triggers molecular and cellular changes in the neuronal body which lead to the activation of regeneration associated genes. In this state, there is a downregulation of neurotransmitter-related proteins, whereas the expression of genes involved in cell survival and neurite outgrowth is increased [1,2]. Simultaneously, the distal segment of the nerve undergoes Wallerian degeneration. The axonal membrane breaks down, cytoskeletal components are degraded and myelin sheaths are dissolved, resulting in the denervation of Schwann cells (SCs). SCs are highly plastic cells that originate from the neural crest and differentiate into two mature phenotypes: myelinating and nonmyelinating [3,4,5]. The maturation of these glial cells occurs in three steps. First, migrating neural crest cells form SC precursors (SCPs), which are multipotent embryonic progenitors. Next, SCPs differentiate into immature SCs. The association of an immature SC with a particular axon determines its fate as a mature SC [6]. In contrast to precursors, mature SC are not dependent on axons for their survival, by establishing an autocrine survival loop [7]. This fact is a major advantage after peripheral nerve injury, avoiding death of SCs after denervation. Mature SCs can be divided in two major subtypes. Myelinating SCs produce myelin sheaths around large nerve fibers and express characteristic myelin markers, such as protein 0 (P0), myelin-associated protein (MAG) and galactocerobroside (Gal-C). The fine tuning of axonal myelination by SCs is controlled by axonal signals, mainly Neuregulin 1 (NRG1) type III [7]. In contrast, nonmyelinating or Remak SCs ensheath smaller unmyelinated axons and maintain some markers present in more immature states, like the cell adhesion molecule L1 and the p75 neurotrophic receptor (p75NTR). After nerve injury, these molecular markers (mostly those related to myelin formation) are downregulated and the denervated SCs acquire a repair phenotype that partially resembles that of immature SCs [8] (Figure 1).

The activation of the transcription factor c-jun triggers this conversion to the repair state [9], which is characterized by numerous changes in gene expression. Many genes related to cell growth, response to external stimuli and neuritogenesis are regulated similarly to immature SCs, reactivating developmental mechanisms. However, an injury-specific program is activated as well, involving also up- or down- regulation of genes related to signal transduction, cell death, immune response, transcriptional regulation, protein transport and metabolism, among others [10,11,12]. The loss of axonal contact also triggers denervated SCs to transiently express NRG1 type I as an autocrine signal to promote SC differentiation and remyelination [13]. Thus, SCs reenter the cell cycle and increase the expression of neurotrophic and chemotactic factors. Consequently, hematogenous macrophages are recruited and, together with SCs, they phagocytose myelin and axonal debris and create a permissive microenvironment to axonal regrowth [14]. Repair SCs proliferate and elongate (2- to 3-fold longer than mature cells and 7- to 10-fold longer than immature SCs) and form processes, in contrast to the shorter and unbranched immature SCs [15]. Following Wallerian degeneration, these enlarged SCs align inside the endoneurial tubules, forming so-called bands of Büngner, where they direct axonal regeneration. 

The ability of peripheral neurons to regenerate after axotomy is crucial for restoring lost functions, but it is usually not sufficient to ensure adequate functional restitution. Successful recovery also requires the specific reinnervation of target organs by appropriate axons, and this is mainly determined by the type and site of lesion [16]. In crush or compressive injuries, where axons are disrupted but connective layers surrounding fascicles (epineurium, perineurium and endoneurial tubules) maintain their continuity, spontaneous regeneration is observed without the need of repair. During the regenerative process, growing axons follow the paths within the endoneurial tubules to reinnervate their original target organs. In contrast, complete transection of the nerve results in a disruption of axons and the connective sheaths. Surgical intervention is then required to reconnect the severed nerve stumps and guarantee that axons can regenerate into the distal nerve. However, even the best microsurgical repair techniques are not enough precise to align endoneurial tubules. Without endoneurial continuity, axons grow at random and are easily misdirected to incorrect targets [17]: motor axons may regenerate towards sensory targets, whereas sensory axons can be misrouted to the muscle. Thus, any type of axon will grow to a peripheral territory that may be different from the originally innervated one. This inaccuracy in reinnervation causes a permanent loss of function that impairs complete recovery, affecting to a greater extent fine functions mediated by large myelinated axons (such as motor control and tactile and proprioceptive sensibilities) than gross functions mediated by thin axons (such as pain and thermal sensibilities).

Despite increasing understanding of the axonal regeneration process, knowledge about specific reinnervation is still scarce. Is there a universal mechanism determining specificity during axonal regeneration? How do neurons respond to environmental cues leading to preferential direction? What is the implication of SCs in this process? Answering these questions will be essential to design strategies that promote specific regeneration and, thus, improve effective recovery after a nerve lesion.

## 2. Current Strategies to Improve Functional Recovery After Peripheral Nerve Injury

Plenty of studies have been performed on treatments to improve axonal regeneration after peripheral nerve injury, either by administering different drugs, adding trophic factors, gene therapy approaches, applying activity-dependent therapies or increasing the intrinsic growth capability of neurons (reviewed in [2,18,19,20,21]). However, the impact of these strategies on accurate reinnervation and functional recovery is usually limited. In general, useful functional recovery is better accomplished by regenerative thin nerve fibers supplying thermal and pain sensitivity and sympathetic efferents than by large nerve fibers, innervating muscles and specialized mechanoreceptors [22]. However, no clear distinction can be made between motor and sensory fibers of the same caliber. 

Interestingly, it has been reported that accelerating the regeneration by genetically increasing the intrinsic capability of neurons to grow could increase motor recovery to a greater extent than sensory recovery. As chronically denervated muscles have a reduced ability to sustain reinnervation by growing axons, faster regeneration was shown to result in enhanced motor recovery [23]. In contrast, activating the mammalian target of rapamycin to increase intrinsic neuronal growth resulted in the promotion of peripheral nerve regeneration, but aberrant reinnervation was also observed [24], indicating that strategies to improve functional recovery should focus on the reinnervation of target organs, rather than on the number of regenerating axons.

Another major line of research has been focused on reparative methods after nerve transection when surgical intervention is essential to guarantee axonal regeneration. After complete disruption of the nerve continuity, suturing the proximal and distal stumps facilitates the growth of the injured axons from the proximal end into the distal stump. SCs have to migrate to guide regenerating axons across a bridge of scar tissue that reconnects the severed nerve ends. SCs use as a track the blood vessels formed by endothelial cells, whose migration is induced by the secretion of VEFG from macrophages that sensed the hypoxia at the newly formed tissue [25].

However, nerve damage often results in tissue loss and nerve retraction, producing a resection. In these cases, direct suture is not allowed, since the gap cannot be repaired without creating tension in the nerve, and the formation of a natural bridge would fail. Therefore, the inclusion of an extrinsic bridge between the stumps is required to ensure regeneration [26]. The current gold standard in clinics is the use of an autologous nerve graft, a nerve segment from the same patient, since the basal lamina and SCs present in these grafts establish a suitable environment for regeneration. However, their use has several drawbacks, including the need for a second surgical step, material limitation, loss of function of donor nerves and the possible mismatch between the caliber of the graft and the injured nerve [27].

Alternatives to autografts include allografts and synthetic nerve conduits. Allografts can be harvested from cadavers and provide a biological scaffold for regeneration. As allografts are immunogenic, they are used in combination with immunosuppression or after a decellularization process. Acellular allografts preserve the basal lamina and tropic factors but lack immunogenic material such as myelin and cells [28]. Thus, these grafts are becoming a valuable alternative to autografts. Segments of skeletal muscle or blood vessels are also an option, since these tissues contain basal lamina as well [26]. On the other hand, conduits offer an easier and cheaper method to repair nerve resections. Axons can regenerate through a nondegradable (e.g., silicone) or degradable (e.g., collagen) tube, although their use is limited to short gaps (up to 10-mm gaps in rats) [29,30]. Despite the advantages of allografts and tubes, none supports regeneration as well as nerve autografts. For this reason, there has been an interest in improving artificial conduits with different strategies. For instance, growth factors, such as NGF, GDNF, BDNF and CTNF, among others, can be loaded into the conduits, stimulating axonal regrowth moderately [31,32]. Cell-based therapies have given more promising results, either by using grafts of SCs or stem cells from different sources (embryonic, neural, mesenchymal, etc.) [30,33,34]. These cells should be able to differentiate into SC-like cells, promoting regeneration by the release of neurotrophic factors and inducing the myelination of regenerated axons. However, functional recovery after nerve transections is never fully accomplished, using either autografts or other alternative conduits. Hence, efforts should be made to enhance not only the quantity and speed of axonal regeneration, but also its quality by increasing specific reinnervation.

## 3. Preferential Motor Reinnervation

Several studies in mammals have described a preference for motor axons to regenerate towards the muscle pathway rather than to the cutaneous pathway, a phenomenon named “preferential motor reinnervation” (PMR) [35,36,37,38]. There are, however, considerable discrepancies in the literature about the mechanisms driving motor axons to their correct path. The most used model to investigate this issue has been the rat or mouse femoral nerve, a mixed nerve that divides into a muscle and a cutaneous branch of similar size. The muscle branch provides efferent and afferent innervation to the quadriceps muscle, whereas the cutaneous branch (the saphenous nerve) gathers sensory information of the skin of the medial lower leg and the hind paw.

Although there is a consensus on the existence of PMR, the accuracy of regenerating motor axons can be modulated depending on the experimental conditions. Brushart described that after transection and repair of the rat femoral nerve proximal to its bifurcation, significantly more motoneurons projected their axons to the muscle branch than to the cutaneous branch both in juvenile and adult rats, even if the repair was intentionally misaligned [36]. Interestingly, this phenomenon was not observed 3 weeks after the nerve repair, when regeneration of motor axons was found at random. In contrast, 8 weeks after injury and repair of the femoral nerve, about 65% of motor axons regenerated through the motor branch. Although significant, this still leaves one-third of the motor neurons misrouting their axons [38]. This phenomenon has also been reported in mice, but only when the transected femoral nerve was repaired with fibrin sealing and not when sutured. Nevertheless, the PMR was highly variable in mice, with animals in which this preference was almost 100% to animals with no preference at all (50%) [39]. Thus, it appears that the size of the nerve and the experimental conditions influence the degree of PMR. 

The PMR phenomenon has given rise to the question of whether the pathway preference originates from signals arriving from the target organs or from the pathway itself. Therefore, in an attempt to better understand the nature of this process, subsequent studies introduced different variations to this model. Contact with target organs can be denied by transecting both distal branches and ligating the proximal stumps, resulting in an absence of preference in the adult rat [37,40] or even a shift of the motor axon preference towards the cutaneous branch in mice [37,41,42]. Moreover, blocking the contact with the muscle but leaving the cutaneous pathway intact has been consistently shown to also reverse this preference of the regenerating motor axons towards the incorrect sensory branch [40,43]. Thus, trophic support or signals coming from the target organs appear to be crucial for PMR [44,45], although other factors, such as the volume of the distal branches [46,47] and the amount of SCs [42,48], also have an influence. Based on these studies, the most accepted hypothesis establishes that there is a hierarchy that determines pathway preference, with muscle contact being the most important factor, followed by the number or density of SCs and the trophic support coming from the skin.

Even though the causative mechanisms of PMR are unknown, evidence strongly indicates that this process occurs in two phases in the femoral nerve. During the first 2 to 3 weeks, motor axons grow into both distal branches, extending multiple regenerating branches [38,40,49,50,51]. Collaterals might prefer the branch with the larger relative amount of basal lamina during this initial phase [47]. Later on, PMR might be achieved by pruning the collaterals from the cutaneous branch while maintaining those in the correct muscular path. Pruning has been proposed to be the result of “sibling bias”, a theory which states that collaterals of the same neuron compete with each other for a limited amount of structural components (membrane, cytoskeleton elements, etc.). Thus, growing axonal branches that receive more trophic support or appropriate cues from the environment will incorporate structural precursors at a higher rate, leaving the supply of these precursors for other collaterals depleted [52]; the question of which specific environmental cues, however, needs to be investigated.

## 4. Motor and Sensory Schwann Cells

It is undeniable that SCs can influence specific regeneration, especially when contact with end organs is prevented [42] or in juvenile rats, that show PMR independently of contact with the end organ [37,49,50]. How they modulate this process is, however, controversial. Some authors propose that SCs maintain a specific molecular identity that axons can recognize during regeneration, favoring PMR. In contrast, others argue that the main effect of SCs is caused by the release of neurotrophic factors. In both cases, the identity or modality of the SCs seems to be important to guide regenerating axons towards their correct target.

### 4.1. Molecular Identity

**Figure 2:**
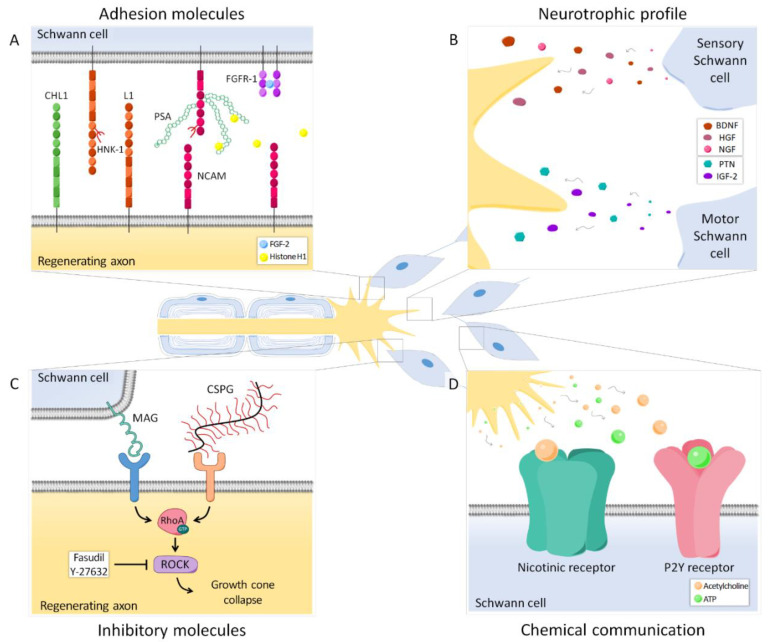
Axon-SC intercommunication during regeneration occurs in different ways and can modulate preferential reinnervation. (**A**) Several adhesion molecules play an important role in guiding axons to their target organs. Polysialic acid (PSA), mainly carried by NCAM, is differentially expressed in the cutaneous and muscle branch of the femoral nerve and its presence can be crucial for specific regeneration. Histone H1 is a PSA ligand that can enhance PMR, whereas FGF-2 promotes regeneration and could also favor specificity, presumably increasing FGFR1 and PSA-NCAM interaction. Other adhesion molecules such as CHL1, L1 and the HNK-1 carbohydrate are also relevant in this process. (**B**) Neurotrophic factors secreted by SCs after nerve injury are important for neuronal survival. Denervated motor and sensory SCs have a specific growth factor expression profile; therefore, regenerating axons receive different signals from the cutaneous and muscle branch of the nerve that can influence axonal pathway choice. (**C**) Some molecules from SCs (MAG) or the extracellular matrix (CSPG) have an inhibitory effect on axonal regeneration. Both MAG and CSPG can activate the small GTPase RhoA and its downstream effector ROCK in axons, resulting in growth cone collapse. Blocking this cascade has a differential effect in motor and sensory axons, thus revealing these inhibitory molecules as candidates for the regulation of PMR. (**D**) SCs can respond to neurotransmitters released by regenerating axons. Particularly, SCs have nicotinic acetylcholine receptors and purinergic P2Y receptors, which allow them to respond to acetylcholine and ATP respectively. This chemical interaction also modulates axon regeneration and specificity.

Extensive evidence suggests that SCs associated with motor axons have different markers and expression patterns than SCs associated with sensory axons (Figure 1 and Figure 2A,B), and that such differences might influence preferential regeneration. The L2/HNK-1 carbohydrate was revealed to be modality-specific since it is strongly expressed in motor-associated SCs, but its expression in sensory SCs is weak [53,54]. Furthermore, its expression is markedly reduced after denervation. Reinnervation of the SCs by motor axons, but not by sensory axons, again induced the expression of this carbohydrate, whereas reinnervation of sensory SCs by motor axons resulted only in weak L2/HNK-1 expression [54]. Thus, these results suggest that SCs previously associated with motor axons retain some of their acquired properties after dedifferentiation, and this selective re-expression of L2/HNK-1 could entail a positive signal to motor axons regenerating in the correct path. On the other hand, neural cell adhesion molecule (NCAM) was shown to be preferentially expressed in the cutaneous branch of the femoral nerve. However, this marker is found mostly in nonmyelinating SCs, specifically, in those associated with sensory but not autonomic fibers [55]. NCAM expression is also reduced after denervation, but axon regeneration normalizes NCAM staining in both muscular and cutaneous branches. Interestingly, sensory axon regeneration using the muscle branch resulted in an upregulation of NCAM in SCs above basal levels [56], suggesting that regenerating motor and sensory axons may influence NCAM expression in SCs.

Altogether, L2/HNK-1 carbohydrate and NCAM can be considered specific markers of muscle and cutaneous nerve branches, respectively. The molecular identity of the SCs and, particularly, the regulation of these markers is determined not only by the classical division between myelinating and nonmyelinating SCs, but also by their association with motor or sensory axons. Thus, while NCAM is mostly found in the cutaneous branch because it is expressed in sensory nonmyelinating SCs, L2/HNK-1 is found in the muscle branch because it contains motor SCs. How this phenotypic heterogeneity influences regeneration is a complex issue, and further research is essential to understanding it.

### 4.2. Neurotrophic Profile

The expression profile of neurotrophic factors also differs between motor and sensory SCs (Figure 2B).

It was described that intact ventral roots predominantly express PTN, VEGF-1 and IGF-1, whereas cutaneous nerves mostly express BDNF, NT-3, HGF and GDNF. After denervation, the expression of these trophic factors also differed between sensory and motor SCs, with PTN and GDNF being strongly upregulated in ventral roots, and NGF, BDNF, VEGF-1, HGF and IGF-1 mainly in cutaneous nerves [57]. The regulation of these factors was further influenced by the modality of the regenerating axons reconnecting the SCs. Reinnervation of cutaneous SCs by sensory axons returned the expression of the upregulated factors to basal levels. However, some of these factors, such as HGF and IGF-1, were not downregulated if motor axons reinnervated the previously sensory SCs. On the other hand, when the ventral root was reinnervated by sensory axons, there was a prolonged expression of GDNF and a transient burst of PTN, whereas matched motor reinnervation had little effect on GDNF and caused a more sustained expression of PTN [57]. 

**Figure 3:**
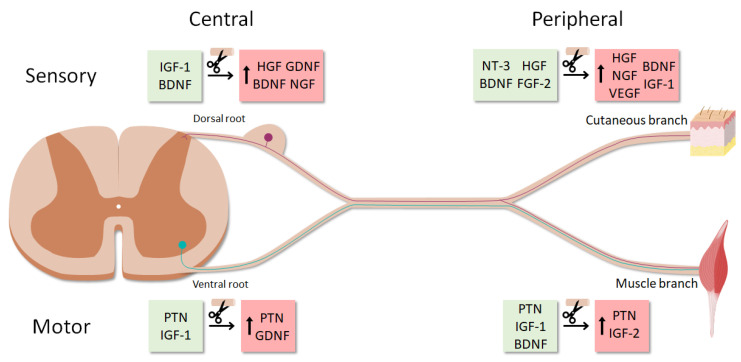
Growth factors are differentially expressed by SCs, depending on their modality and their location in the central-peripheral axis. After denervation, the regulation of neurotrophic factor expression also varies between SC subtypes. For instance, GDNF is mainly upregulated in dorsal and ventral roots, whereas PTN is highly upregulated in denervated motor SCs, both central and peripheral. Denervated sensory SCs in the peripheral nerve strongly upregulate several factors, among which HGF, BDNF and NGF are the most distinctive.

Recently, the SC neurotrophic profile was found to vary not only by the modality of the associated axons, but also by their central-peripheral location [58]. In an elegant study, Brushart et al. used diverse surgical preparations to separately study the denervation of SCs in the dorsal root, cutaneous nerve, cutaneous unmyelinated nerve, ventral root and muscle nerve (efferent and afferent). This made it possible to complete the trophic expression patterns previously established by Höke et al. only in the ventral root and cutaneous nerve [57] (Figure 3). GDNF was revealed as a “central” factor, since it was predominantly upregulated in both denervated ventral and dorsal roots compared to peripheral SCs.

On the other hand, IGF-1 and VEGF were limited to the periphery, where their expression was significantly higher in cutaneous than in muscle nerves. In agreement with previous studies, PTN was found to be motor-specific [59], being expressed mainly in ventral roots and muscle efferents. Finally, HGF, BDNF and NGF were confirmed as sensory-specific for their marked upregulation in denervated cutaneous nerve and dorsal root [58]. It is worth noting that these differences might not arise from the motor-sensory SC dichotomy exclusively, since ventral roots contain motor exit point glia [60]. These cells differ from SCs in developmental origin and function, which may partially explain the neurotrophic profile variation among the central-peripheral axis. 

Does this characteristic pattern of neurotrophic expression play a role in PMR? This issue has been more difficult to solve, since some modality-specific factors do not necessarily promote the regeneration of their modality-matched axons. A clear example is BDNF, a trophic factor expressed by denervated sensory SCs [57,58], which has been also described to specifically promote neurite outgrowth of motor neurons in vitro [61,62,63]. Although modestly, this factor also selectively enhanced motor regeneration in vivo [62,63,64]. Furthermore, the in vitro capabilities of some trophic factors to increase the neurite growth of either sensory or motor neurons are reduced when these factors are tested in vivo [62,63,64]. The discrepancies between in vitro and in vivo results when testing the ability of trophic factors to promote axonal growth highlight the limitation of the in vitro studies, in which cells grow in nonphysiological conditions. DRG explants and organotypic spinal cord slices, by maintaining the cytoarchitecture of the tissue, can be considered better in vitro models than dissociated cultures. However, even in those organotypic cultures, the addition of serum to the medium to promote neuronal survival can directly affect the response of neurons to trophic factors. In fact, it was shown that serum decreases the production of autocrine survival signals in postnatal SC cultures, thus altering their physiological response to denervation [65]. Therefore, in neuronal cultures where SCs are present, dysregulation of the normal responses of these cells can also contribute to differences with the findings observed in vivo. 

### 4.3. Gene Expression

After nerve injury, gene expression in SCs is dramatically changed. Hundreds of genes involved in cell cycle, cell proliferation, immune cell function, synaptic structure and neuron function, among others, are differentially regulated after axotomy [8,10,11,12]. Interestingly, additional to the neurotrophic factor expression, SCs from the muscle and cutaneous branches of the femoral nerve also show several other genes with different expression patterns. For instance, some genes related to motor nerve myelination and signaling, such as neurofilament light polypeptide (Nefl) and protein kinase C iota (Prkci), were found to be upregulated in the motor branch in comparison to the sensory branch of the rat femoral nerve. In contrast, the cutaneous nerve had a high expression of genes involved in promoting sensory nerve myelination and maturation, such as neuroligin (Nlgn1) and myelin basic protein (Mbp) [66], and also of genes that regulate proliferation and migration (nap1l1, dok4, lpp, mmp-9, l1cam) [67]. Importantly, it was demonstrated that the expression pattern of specific markers of motor and sensory SCs was dysregulated in culture. Thus, the motor markers Nefl, PTN and Prkci were downregulated in motor SCs or upregulated in sensory SCs when cultured in vitro. On the other hand, the differential overexpression of Mbp in the sensory branch of the femoral nerve in vivo was lost in vitro, where both motor and sensory SCs overexpress this protein. This dysregulation could be, in part, related to the absence of neurons in the culture [66].

### 4.4. Phenotypic Memory

It has been suggested that the phenotypic identity of motor and sensory SCs can be maintained through several cell divisions, since the pattern of growth factor expression of SCs from the ventral and dorsal roots was partially preserved in vitro for 2 weeks [58]. This is in agreement with in vivo studies that showed that prolonged reinnervation of the ventral root by cutaneous axons shifted the SCs phenotype to a more cutaneous identity (high BDNF expression) while retaining evidence of their original motor phenotype (high GDNF expression) [57].

The phenotypic memory of the SCs after denervation might play a role in PMR, since graft modality used to repair a nerve resection influences the outcome of axonal regeneration. While some studies could not find improved motor regeneration when the femoral or the facial nerve were repaired with modality-matched grafts [68,69], others found that motor axons from the sciatic and tibial nerve regenerated better through a ventral root or a motor nerve than through a sensory graft [57,70,71]. The selective effect may be partially sustained in predegenerated grafts. Thus, when a segment of ventral or dorsal root predegenerated one week in vitro was allotransplanted in rat sciatic nerve, the regeneration of motor axons was promoted early on by the motor graft, whereas reinnervation of sensory pathways was increased by the sensory graft [72]. The use of shortly degenerated grafts may be advantageous over dissociated SCs maintained longer in culture, since the expression of modality markers, such as L2/HNK-1, declines with time in denervated SCs [55,72].

This is also in line with a recent study that demonstrated that cultured neurons extend significantly longer neurites towards their phenotype-matched SCs. Nonetheless, if these muscle and cutaneous nerve-derived SCs were previously treated with GDNF, the phenotype mismatch was overcome, and both motoneurons and DRG neurons elongated neurites to the same extent, independently of the cocultured SC origin [73]. The positive effect of GDNF in regeneration was confirmed in vivo through its sustained delivery in denervated nerves. After 3 months of denervation, the distal stump of the tibial nerve was sutured to a freshly cut peroneal nerve. Five weeks later, motoneurons had regenerated more in the group with GDNF delivery, whereas this factor did not affect sensory neuron regeneration [74]. A similar enhancement of motor axon regeneration was found after treating a denervated saphenous nerve with GDNF and grafting it to the muscle branch of the femoral nerve. Motoneurons were able to regenerate in the pretreated sensory grafts to the same extent as in the positive control (denervated quadriceps nerve graft) and significantly better than in sensory grafts without the GDNF treatment [75]. The mechanism by which this trophic factor presumably enhances specific regeneration is unclear. GDNF seems to promote SC differentiation to their original phenotype [75,76], but this would increase the effect of phenotype mismatch and, thus, most likely hinder motor axon regeneration. This exogenous GDNF also stimulates endogenous GDNF production in SCs [73,75] by a positive-feedback mechanism, and perhaps this overall increase in GDNF and other trophic factors, such as BDNF [75,76], is responsible for the enhanced axonal regeneration.

## 5. Adhesion Molecules

### 5.1. Polysialic Acid

Interactions between regenerating axons and SCs through adhesion molecules play an important role in guiding axons to their target organs (Figure 2A). One of these relevant molecules is polysialic acid (PSA), an anionic glycan that is mainly carried by NCAM in the cell surface of neurons and SCs. Due to its negative charge, PSA traps water between cells, thus increasing membrane to membrane distance and reducing intercellular interactions [77]. It has been shown that, in normal conditions, PSA is expressed in the cutaneous branch but not in the muscle branch of the femoral nerve [78,79], probably following the pattern of NCAM expression in nonmyelinating sensory SCs. However, after axotomy, this glycan is highly upregulated in both branches [78,79]. A lack of PSA and NCAM in transgenic mice NCAM^−/−^ resulted in the absence of PMR in the femoral nerve 6 weeks after transection. Removing PSA enzymatically in wild type mice had the same effect, strongly suggesting a major role of this molecule in specific regeneration. Sprouting of regenerating axons was maintained more distally in NCAM^−/−^ than in wild type mice, which indicates that misdirected motor axon collaterals cannot be effectively pruned in these animals. Since PSA decreases adhesive interactions in the distal stump mediated by laminins and L1, the lack of this glycan might result in increased growth-related signals that surpass the pruning cues [78].

In agreement with these results, it was demonstrated that some nerves that do not upregulate PSA after axotomy, such as the obturator and genitofemoral, do not show PMR either. Furthermore, electrical stimulation was able to upregulate this adhesion molecule in the femoral and obturator, but not in the genitofemoral nerve. In both nerves with increased PSA, there was a parallel enhancement of PMR, whereas the genitofemoral did not exhibit motor preference in any condition, supporting the importance of PSA [80]. Despite the undeniable relevance of PSA in specific regeneration, this glycan was recently demonstrated to be nonessential for PMR. When the obturator is sutured to the femoral nerve, but the cutaneous branch is ligated, denying access to the skin, motoneurons regain the ability to regenerate preferentially using the muscle branch [79]. 

Although PSA might not be a major determinant for specific regeneration, diverse approaches targeting its pathway achieved a modest effect enhancing PMR and slightly clarified its potential mechanism of action. Using organotypic cultures of the spinal cord and DRG explants, the low molecular weight fibroblast growth factor 2 (FGF-2) was found to be a promising factor selectively promoting motor neurite outgrowth. Its effects were shown to be PSA-dependent, since enzymatically blocking PSA with endoneuraminidase reverted the positive effect of FGF-2 on neuritogenesis. Moreover, PSA-NCAM was found to be interacting with FGFR-1, the main receptor of FGF-2 in the nervous system. Thus, the authors hypothesized that FGF-2 favors interactions between FGFR-1 in SCs and PSA-NCAM in motoneurons [61]. In vivo, a neural guide containing SCs overexpressing FGF-2 to repair the resected sciatic nerve promoted both motor and sensory axon regeneration, although motor regeneration was moderately favored [81]. Therefore, it is not clear whether FGF-2 can enhance PMR in vivo. Another study pointed out that the NCAM-FGFR interaction could be crucial to mediate the myelination-promoting effect of a PSA mimicking peptide. However, the exogenous application of this PSA mimic in a femoral nerve model did not have any effect on specific regeneration [82]. Finally, extracellular histone H1 was revealed as a novel PSA ligand. Applied in culture, it increased the neurite length of cerebellar neurons, as well as the process length and proliferation of SCs through a PSA-dependent mechanism. In vivo, the application of histone H1 at the injury site during a femoral nerve transection and repair resulted in enhancement of PMR compared to the control group, treated with PBS [83]. Thus, different studies have implicated the PSA pathway in preferential regeneration, but there is a need to extend this knowledge and clarify the exact role of this adhesion molecule in the specificity process.

### 5.2. Other Adhesion Molecules

The enhancement of PMR has been tested with other adhesion-related molecules, such as the HNK-1 carbohydrate, the neural cell adhesion molecule L1 or the close homolog of L1 (CHL1). Since L2/HNK-1 was shown to be differentially expressed by motor axon-associated SCs [54], an HNK-1 mimic was proposed as a candidate to favor specific regeneration. This was confirmed by applying the HNK-1 mimic in the transected and repaired femoral nerve of mice; after 3 months, it was observed that motoneurons had regenerated more through their correct pathway compared to control groups [84]. Similarly, PMR was moderately improved using function-triggering antibodies to L1, a glycoprotein upregulated in regenerating axons and SCs after axotomy [85]. The role of this molecule is, however, not clear, since genetic ablation of L1 also enhanced specific motor axon regeneration, allegedly as a result of an increased SC proliferation [86]. CHL1 is another adhesion molecule that can establish homophilic or heterophilic interactions, leading to a variety of actions [87,88]. This molecule has been shown to be upregulated in regenerating axons as early as 1 day after axotomy, whereas SCs expressed it at later time points. A lack of CHL1 in transgenic mice decreased the preference of motor axons to regenerate through the muscle branch of the femoral nerve, indicating that this molecule influenced PMR. The authors hypothesized that heterophilic interactions between neuronal CHL1 and other molecules could facilitate regrowth during the first 2 days, and thus, increase PMR, possibly through a sema3A-dependent mechanism [89]. 

Altogether, evidence suggests that axon-SC intercommunication has a relevant role in the regulation of specific regeneration. So far, direct contact through adhesion molecules has received substantial attention, although the mechanisms and the exact interactions determining their influence in PMR are still poorly understood. Nonetheless, there are other axon-SC communication pathways, such as neurotransmitters released by growth cones (Figure 2D). SCs have nicotinic acetylcholine receptors and P2Y receptors that allow them to respond to acetylcholine and ATP, respectively. This chemical communication has been shown to also modulate specificity and should be considered to further understand this process [90].

## 6. Inhibitory Molecules

During Wallerian degeneration, myelin breakdown results in the exposure of growth inhibitory molecules such as myelin-associated glycoprotein (MAG) [91,92]. In the peripheral nervous system, MAG is present in the periaxonal region, where it plays an important role in the maintenance of normal interactions between myelinating SCs and their axons [93]. The blockade of this glycoprotein by systemic administration of antibodies has been shown to increase PMR dramatically in mouse [94]. In contrast, specific regeneration of sensory neurons was either decreased (after 4 weeks) or unaffected (after 6 weeks), suggesting that MAG might differentially influence motor and sensory axon regeneration [94].

Other inhibitory molecules which are relevant in peripheral nerve regeneration are chondroitin sulfate proteoglycans (CSPGs), extracellular matrix components that are upregulated after nerve injury [95]. In the case of CSPGs, their removal using the enzyme chondroitinase ABC also improved regeneration [74,96], although it did not have a differential effect on motor versus sensory neurons [74]. Both MAG of SCs and CSPGs of the extracellular matrix induce growth cone collapse through the activation of the small GTPase RhoA and its downstream effector Rho-kinase (ROCK) in regenerating neurons [97,98] (Figure 2C). Importantly, it was recently demonstrated that motor and sensory neurons respond differently to the pharmacological inhibition of this pathway. When neurons were cultured in the presence of CSPGs and treated with the chemical compound Y-27632, a ROCK inhibitor, motor neurons could extend longer neurites than without the inhibitor, whereas sensory neurite outgrowth was not significantly enhanced. This differential effect was confirmed in a nerve crush model in vivo, in which only motor axons demonstrated an improvement in regeneration after the administration of the drug [99].

Thus, inhibitory molecules influence regeneration after nerve injury and, due to their differential effect on motor and sensory neurons, might also participate in the regulation of preferential reinnervation.

## 7. Electrical Stimulation

Electrical stimulation (ES) has been repeatedly demonstrated to improve axonal regeneration after nerve injury (for review see [100]). Al-Majed et al. showed that 1 h of low-frequency stimulation (20 Hz) proximal to the repair site was enough to accelerate PMR in the femoral nerve of rat [101]. Later, a similar effect was demonstrated for sensory neurons as well [102].

This accelerated axonal regeneration was found to correlate with an early upregulation of BDNF and its receptor, trkB [103], which is mediated by the entrance of extracellular calcium and Erk-dependent signaling pathways [104] in the neuron. In fact, the genetic ablation of trkB abolished the effect of ES in preferential regeneration, suggesting an essential role of BDNF/trkB signaling mediating this process [105]. Importantly, this trophic factor was shown to regulate the expression of the HNK-1 carbohydrate, a crucial molecule in SC molecular identity [105]. Similarly, ES induced the premature upregulation of regeneration associated genes such as growth-associated protein 43 (GAP-43) in motor and sensory neurons [106,107], a process that might be also linked to BDNF increased expression. Taken together, these changes allow motoneurons to regenerate through the suture line earlier than they would without stimulation, thus reducing the regeneration “staggering” period that normally occurs after nerve injury [108]. Nevertheless, understanding how these alterations can improve specific reinnervation is more challenging. Two possible mechanisms were proposed by Brushart et al. [102]: (1) The accelerated regeneration caused by ES results in more axons contacting the muscle at earlier time points and, therefore, there is a faster pruning of misdirected collaterals; (2) Some modality cues in the nerve are lost during Wallerian degeneration, but the enhanced regeneration allows axons to reach more of these signals that might favor specificity. Indeed, other procedures that accelerate the regeneration rate, like nimodipine treatment [109] and surgical repair with predegenerated grafts [38], have been shown to reduce misdirected reinnervation in the facial and femoral nerves, respectively.

Most studies have been focused on the effect of ES on neurons, but the application of electrical stimuli to the nerve affects both neurons and non-neuronal cells, i.e., SCs, fibroblasts and endothelial cells. Recently, it was shown in vitro that ES influenced SC morphology and orientation. These prestimulated SCs were able to promote neurite outgrowth when cultured with unstimulated neurons. Increased neuritogenesis was also achieved using only the conditioned medium from the electrically stimulated SCs [110], a fact that suggests that ES induced the expression of neurotrophic factors in SCs. Indeed, NGF release was found to be strongly increased in cultured SCs after ES [110,111]. Furthermore, an in vivo study showed that ES following sciatic nerve section and repair enhanced motor and sensory reinnervation and accelerated the mRNA expression of BDNF and GDNF in DRG and of BDNF and NT-3 in the ventral horn [112]. However, the question of whether SCs modulate specific regeneration after ES remains unexplored.

## 8. Conclusions

After peripheral nerve injury, guiding axons back to their original target organ remains a major problem hindering complete functional recovery. Axonal regeneration is a complex process in which several components are involved. Hence, understanding the interactions between axons and their environment is essential to improve specific regeneration. Among the several factors influencing this process, target organs are perhaps the most relevant, either by their supply of trophic factors, or by direct contact with axons after reinnervation, which might determine the pruning of misdirected collaterals. However, many other factors have emerged as candidates that regulate preferential regeneration. SCs show modality-specific characteristics that can influence their interaction with regenerating axons. They are a major source of neurotrophic factors in the nerve, but they can also undergo direct communication through adhesion molecules, such as PSA or HNK-1, or even respond to neurotransmitters released by regenerating growth cones. Many of these specific cues and interactions can be modulated to achieve better and more accurate regeneration after axotomy and, thus, improve the functional recovery of patients suffering nerve injuries.
